# Unlocking Drug Resistance in Multiple Myeloma: Adipocytes as Modulators of Treatment Response

**DOI:** 10.3390/cancers15174347

**Published:** 2023-08-31

**Authors:** Maria Ochiai, Sara Fierstein, Farouq XsSali, Nicholas DeVito, Laura R. Purkey, Rebecca May, Abraham Correa-Medina, Mary Kelley, Thomas D. Page, Kathleen DeCicco-Skinner

**Affiliations:** Department of Biology, American University, 4400 Massachusetts Ave, NW, Washington, DC 20016, USA

**Keywords:** multiple myeloma, adipocyte, obesity, resistance, P-glycoprotein, BMI

## Abstract

**Simple Summary:**

Worldwide obesity has nearly tripled in the last fifty years. Obesity is a risk factor for multiple myeloma, contributing to both an increased risk of multiple myeloma development and poor survival outcomes. Obesity is associated with the accumulation of fat cells (adipocytes) in bone marrow. These adipocytes can interact with, and modify the behavior of, nearby multiple myeloma cells. In this report, we found that adipocytes activate drug-resistance mechanisms in multiple myeloma cells, thus decreasing the effectiveness of anti-cancer drugs. The effects were more pronounced when adipocytes were derived from overweight or obese patients. Thus, we suggest a possible underlying mechanism by which obesity can allow for treatment failure and/or disease progression in multiple myeloma patients.

**Abstract:**

Multiple myeloma (MM) is an incurable hematological malignancy characterized by the clonal proliferation of malignant plasma cells. Despite the development of a diverse array of targeted drug therapies over the last decade, patients often relapse and develop refractory disease due to multidrug resistance. Obesity is a growing public health threat and a risk factor for multiple myeloma, although the mechanisms by which obesity contributes to MM growth and progression have not been fully elucidated. In the present study, we evaluated whether crosstalk between adipocytes and MM cells promoted drug resistance and whether this was amplified by obesity. Human adipose-derived stem cells (ASCs) from nineteen normal (BMI = 20–25 kg/m^2^), overweight (25–30 kg/m^2^), or obese (30–35 kg/m^2^) patients undergoing elective liposuction were utilized. Cells were differentiated into adipocytes, co-cultured with RPMI 8226 or U266B1 multiple myeloma cell lines, and treated with standard MM therapies, including bortezomib or a triple combination of bortezomib, dexamethasone, and lenalidomide. We found that adipocytes from overweight and obese individuals increased cell adhesion-mediated drug resistance (CAM-DR) survival signals in MM cells, and P-glycoprotein (P-gp) and multidrug resistance-associated protein (MRP) drug transporter expression. Further, co-culture enhanced in vitro angiogenesis, MMP-2 activity, and protected MM cells from drug-induced decreases in viability. In summary, we provide an underlying mechanism by which obesity can impair the drug response to MM and allow for recurrence and/or disease progression.

## 1. Introduction

Multiple myeloma (MM) is an incurable hematologic malignancy with nearly 36,000 cases diagnosed in the United States in 2022 [1]. Although overall survival has tripled in recent decades, MM remains an incurable disease with a median life span of 5–8 years, depending on the patient’s age and treatment regimen [2,3].

Obesity, as defined by a body mass index (BMI) ≥ 30 kg/m^2^, is increasing at an alarming rate in the U.S. [4]. Currently, 73.6% of all people in the U.S. over the age of 20 are overweight (BMI of 25–30 kg/m^2^) or obese [5]. Obesity is associated with the development of thirteen different forms of cancer, including multiple myeloma [6]. Overweight and obese individuals have an increased risk of developing MM and a significantly higher mortality rate after diagnosis [7,8,9] However, the biological link between obesity and MM etiology has not yet been fully characterized.

MM is characterized by the clonal expansion of malignant plasma cells in the bone marrow [10]. The bone marrow niche contains various cell populations capable of communicating with MM cells and contributing to the pathogenesis of this disease [11,12,13]. The most predominant cell type in bone marrow is the adipocyte, which occupies 50–70% of bone marrow volume in adulthood, increasing to greater than 70% in elderly populations [14,15]. Adipocytes are dynamic cells that can alter their endocrine, cytokine, and signaling pathways in response to obesity [16,17]. While there has been increasing interest in understanding the crosstalk between adipocytes and cancer, the role of adipocytes in MM growth, survival, and progression is a rather underexplored area of research. We previously reported that adipocytes can support MM cell viability, adhesion, and angiogenic potential [18]. Others have reported similar MM/adipocyte reciprocal relationships, showing that adipocytes can increase MM cell chemotaxis, tumor growth in bone, bone microenvironment, and that MM can induce bioenergetic changes in adipocytes [19,20,21].

The introduction of new therapeutic agents for MM in the last twenty years and the optimization of treatment regimens have significantly improved the survival of MM patients [22,23]. Typically, physicians have adopted a multidrug approach in combating multiple myeloma, using a combination of proteasome inhibitors such as bortezomib or carfilzomib, angiogenic inhibitors/immunomodulatory drugs including thalidomide, lenalidomide, and pomalidomide, and glucocorticoids such as dexamethasone [24]. Despite significant advances in treatment, relapsed and refractory patients with MM are a vulnerable population with unmet medical needs [2]. This relapse is typically associated with antineoplastic drug resistance [25,26]. Unfortunately, once patients become resistant to proteasome inhibitors and immunomodulatory drugs, they have a very poor outcome [27].

In MM, several different intrinsic and extrinsic mechanisms of drug resistance explain patient relapse [25,26,28,29]. Two common modes of drug resistance in cancer are multidrug resistance (MDR) and cell adhesion mediated drug resistance (CAM-DR) [26]. The MDR mechanism involves overexpression of the ATP-dependent efflux pumps P-glycoprotein (P-gp) and multidrug resistance-associated protein 1 (MRP1) [30]. This increased efflux can reduce the intracellular concentration of antineoplastic drugs, thus conferring drug resistance. Cell adhesion mediated drug resistance (CAM-DR) is associated with the activation of nuclear factor kappa B (NFκB), alteration of intracellular drug targets, integrin-mediated adhesion, and resistance to drug-induced apoptosis [31,32,33]. Activation of both processes has been reported in drug-resistant MM cells [31,34,35,36,37]. However, the contribution of adipocytes to these drug-resistance mechanisms and the relationship between patient BMI and MDR/CAM-DR pathway activation is unknown.

In this paper, we evaluated whether adipocytes differentiated from adipose-derived stem cells (ASCs) of normal, overweight, or obese individuals could promote resistance to bortezomib or a triple-drug combination of bortezomib, lenalidomide, and dexamethasone in MM cells, and the extent to which this was mediated through P-glycoprotein (MDR1) and/or CAM-DR pathways. We found that adipocytes regulate drug response in MM cells and may serve as a barrier to effective cancer treatment. Further, the effect was more pronounced when MM cells were co-cultured with differentiated ASCs from patients with BMIs in the overweight or obese category, suggesting a possible underlying mechanism by which obesity can allow for recurrence and/or disease progression.

## 2. Methods

### 2.1. Multiple Myeloma Cell Lines

Human MM cell lines, RPMI 8226 and U266B1 (ATCC; Manassas, VA, USA), were cultured in RPMI 1640 medium supplemented with 1% GlutaMAX, 1% PenStrep (Thermo Fisher Scientific; Waltham, MA, USA), and 10% fetal bovine serum (FBS) (Peak Serum; Wellington, CO, USA). The cultures were incubated at 37 °C and 5% CO_2_.

### 2.2. Adipose-Derived Stem Cell Culture and Differentiation to Adipocytes

Human adipose-derived stem cells (ASCs) isolated from abdominal adipose tissue of nineteen healthy non-diabetic donors undergoing elective surgery were obtained from Zen-Bio, Inc. (Research Triangle Park, NC, USA). All ASCs, and subsequently differentiated adipocytes, were grouped by BMI into one of three categories: normal (BMI = 20–24.9 kg/m^2^), overweight (25–29.9 kg/m^2^), or obese (>30 kg/m^2^; Table 1). ASCs were grown in tissue-culture treated flasks (Thermo Fisher Scientific; Waltham, MA, USA) using DMEM (Thermo Fisher Scientific; Waltham, MA, USA) supplemented with 1% GlutaMAX, 1% PenStrep, and 10% FBS. The ASCs were passaged at least twice before they were moved to 6-well plates and differentiated into adipocytes. Adipocyte differentiation media consisted of DMEM media supplemented with 1% GlutaMAX, 1% PenStrep, 10% FBS, 0.2 mM indomethacin, 1 µM dexamethasone, 10 µM insulin, and 0.5 mM isobutylmethylxanthine (IBMX) (Sigma-Aldrich; St. Louis, MO, USA). Full differentiation occurred by day 14, at which point adipocytes were co-cultured with MM cell lines.

### 2.3. Adipocyte and MM Co-Culture

**Figure 1:**
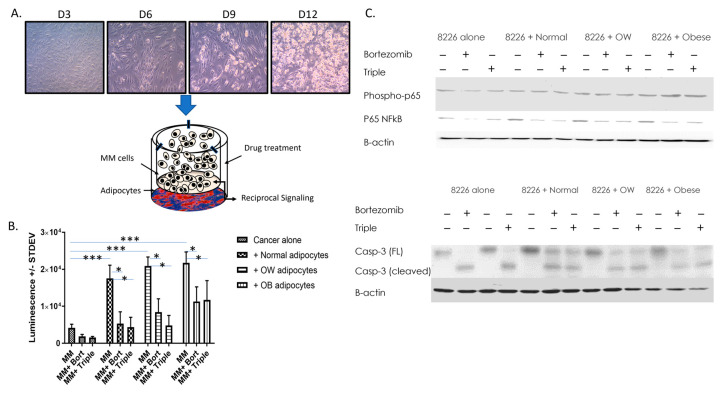
Adipocytes increase MM cell viability and resistance to drug-induced decreases in viability. (**A**) ASCs from normal, overweight, or obese patients were differentiated into adipocytes and co-cultured with RPMI 8226 or U266B1 MM cells. Bortezomib alone or bortezomib, lenalidomide, and dexamethasone in combination was added to some wells. MM cells +/− drugs cultured without adipocytes were used as a control. After 15 min (for phosphorylated proteins) or 24 h, cells were collected and cells or protein used for analysis. (**B**) MM cell viability was measured in control or drug-treated MM cells cultured alone or in the presence of adipocytes from patients in different BMI categories. N ≥ 4 patients/BMI category; * *p* ≤ 0.05, *** *p* ≤ 0.001. (**C**) Western analysis of phospho-p65, total p65, and caspase-3 in MM cells +/− drugs cultured with or without the presence of adipocytes. Uncropped WB images are shown in Figure S1.

For co-culture, 0.4 µm polyethylene terephthalate (PET) hanging cell culture inserts containing 3 million MM cells/insert (EMD Millipore; Chicago, IL, USA) were placed into 6-well plates containing differentiated adipocytes from individual normal, overweight, or obese patients, allowing MM and adipocyte cells to communicate but remain as separate cell populations (Figure 1A). Additional plates containing only MM or only adipocyte cells (non-co-cultured) were used as controls. Inserts containing MM cells were treated with vehicle only, 10 nM bortezomib, or a combination of 10 nM bortezomib, 1 µM dexamethasone, and 1 µM lenalidomide. After 15 min (for phosphorylated proteins) or 24 h of co-culture, cells and conditioned media were collected from treated and untreated MM cells.

### 2.4. Cell Viability Assay

To measure cell viability, the CellTiter-Glo Assay was used (Promega; Madison, WI, USA) per the manufacturer’s instructions. Untreated or treated, co-cultured or non-co-cultured MM cells in 25 μL media were transferred to opaque-walled 384-well plates in quadruplicate and mixed with 25 μL of CellTiter-Glo Reagent. Control wells containing media with no cells were used to determine background luminescence. After cell lysis, followed by room temperature incubation, luminescence was measured using a Molecular Devices Filtermax F5 plate reader. Viability assays were repeated three times.

### 2.5. Protein Isolation/Western Blotting

Total protein lysates were prepared from co-cultured or non-co-cultured MM cells using RIPA reagent containing Halt protease/phosphatase inhibitor (Thermo Fisher Scientific; Waltham, MA, USA) per the manufacturer’s protocol. Proteins were quantified using a BCA protein assay kit (Thermo Fisher Scientific; Waltham, MA, USA). 25 ug of total protein lysate was separated using 4–12% Tris-Glycine gels (Thermo Fisher Scientific; Waltham, MA, USA). After electrophoresis and transfer to PVDF membrane, membranes were blocked in 5% non-fat dry milk. Primary antibodies were added overnight at 1:1000 concentration. The following antibodies purchased from Cell Signaling Technology (Danvers, MA, USA) were used in this study: BCL-xL (Cat #2764), NFκB p65 (Cat #8242), caspase-3 (Cat #9662), caspase-8 (Cat #9746), caspase-9 (Cat #9502), P-glycoprotein (Cat #13342), MRP1 (Cat #72202), phospho-p65 (Cat #3033), and B-actin (Cat #4970). Anti-rabbit (Cat #7074) or anti-mouse (Cat #7076) HRP secondary antibody was added in a 1:2000 dilution (Cell Signaling Technology). West Dura chemiluminescent substrate (Thermo, Rockland, IL, USA) was applied and images were visualized using a ChemiDoc-It imaging system (Analytik-Jena, Upland, CA, USA). All Western blot bands were quantified using NIH Image J and normalized to B-actin. All Western blots were repeated a minimum of three times. Uncropped Western blot images and respective densitometry graphs are shown in Supplementary Data (Figures S1–S4)

### 2.6. Quantitative PCR

SuperScriptR III PlatinumR SYBR Green Two-Step qPCR Kit w/ROX was used per manufacturer’s instructions (Life Technologies; Grand Island, NY, USA) using 1 μg of purified total RNA as template for amplifications. qPCR was performed on an Mx3005P QPCR System (Agilent Technologies; Santa Clara, CA, USA). Threshold cycle values were calculated with MxPro QPCR software (v. 4.10) using the Pfaffl method and dissociate curves for each reaction were checked to confirm that only a single PCR product was obtained. Threshold cycle values were normalized with GAPDH and relative expression was calculated comparing differences between MM samples. An unpaired *t*-test between triplicates was used to determine level of significance. All experiments were repeated a minimum of three times. The specific oligonucleotide primers obtained from ThermoFisher (Waltham, MA, USA) are listed in Table 2.

### 2.7. Endothelial Cell Tube Formation Assay

U266B1 cells were cultured alone or co-cultured with adipocytes from normal, overweight, or obese patients. Some co-cultures were treated with bortezomib or a triple-drug combination (bortezomib/lenalidomide/dexamethasone). Conditioned media were removed 24 h post-drug treatment and filtered to remove cellular debris. Tube formation using 1 × 10^5^ 3B11 endothelial cells exposed to conditioned media from co-cultured cells was performed as previously described [38,39]. The tube network was allowed to grow for 6 h before paraformaldehyde fixing and imaging using a fluorescence microscope (Olympus; Center Valley, PA, USA). The tube network displaying the number of nodes/meshes/segments was quantified using NIH ImageJ with the Angiogenesis Analyzer Plugin [39].

### 2.8. Zymography

U266B1 or RPMI 8226 MM cells were cultured alone or co-cultured with adipocytes from normal, overweight, or obese patients. Some co-cultures were treated with bortezomib or a triple-drug combination (bortezomib/lenalidomide/dexamethasone). Conditioned media were removed 24 h post-drug treatment and filtered to remove cellular debris. To measure MMP-2 and MMP-9 enzymatic activity, zymography was conducted using 10% Tris-Glycine gels containing 0.1% gelatin (Thermo Fisher Scientific; Waltham, MA, USA). Conditioned media were mixed with B-mercaptoethanol free 4X loading buffer and electrophoresed at 125 V for 90 min in Tris-Glycine/SDS running buffer. After renaturation, developing solution was applied to gels, gels were washed in deionized water, stained in SimplyBlue SafeStain (Thermo Fisher Scientific; Waltham, MA, USA), and destained in deionized water. Densitometry was performed using NIH Image J. All zymograms were repeated three times. Uncropped zymogram gels are shown in Figure S5.

### 2.9. Oil Red Staining

Intracellular neutral lipid accumulation was visualized using Oil Red O staining. Oil Red O was prepared in isopropanol. After removal of media, cells were fixed in 10% formalin for 1 h, washed in water and 60% isopropanol, and stained with 60% Oil Red O working solution for 20 min. Cells were rinsed four times with water. Hematoxylin was applied for 1 min as a counterstain. Cells were washed an additional 3 times with water and immediately visualized/photographed with a phase-contrast microscope.

### 2.10. Statistical Analysis

Data were tested for normality, model assumptions were checked, and analyzed with SPSS software. Tube formation assays, zymography, qPCR, and proliferation studies examining genotype and drug effects were analyzed through two-way ANOVA with Tukey’s post-hoc test. Significance for all analyses was assumed at a *p*-value of 0.05 or less. Significance values of *p* < 0.05 are indicated in figures with a single asterisk *, *p* < 0.01 with a double asterisk **, and *p* < 0.001 with a triple asterisk ***.

## 3. Results

### 3.1. Adipocytes Increase Cell Proliferation and Resistance to Drug-Induced Decreases in Viability in MM

A cell viability assay was performed on RPMI 8226 cells cultured alone or co-cultured with adipocytes differentiated from ASCs from normal, overweight, or obese BMIs (Figure 1). A subset of cells was treated with bortezomib or a triple-drug cocktail (dexamethasone, lenalidomide, and bortezomib). When grown in the presence of normal, overweight, or obese adipocytes, cell viability of RPMI 8226 cells increased 4.2-fold, 5-fold, and 5.2-fold respectively (*p* < 0.001). Additionally, cells co-cultured with higher BMIs were less responsive to bortezomib or triple drug-induced decreases in viability. Bortezomib inhibited the growth of RPMI 8226 cells co-cultured with normal adipocytes 69.7%, but only inhibited RPMI 8226 co-cultured with overweight or obese adipocytes, 59.7% and 48.0%, respectively (*p* < 0.001). Similarly, the triple cocktail was able to inhibit MM growth 75% in MM co-cultured with normal adipocytes, but only 46% in MM cells co-cultured with obese adipocytes (*p* < 0.001). The effect of co-culture (*p* < 0.001), drug (*p* < 0.001), and the interaction of both (*p* < 0.014) was significant. Further, phospho-p65, total p65, and cleaved (active) caspase-3 levels were measured in non-co-cultured and co-cultured MM cells +/− drugs. Phospho-p65 was increased two-fold in MM cells co-cultured with adipocytes from obese individuals. Total p65 was increased 2.5-, 2.4-, or 1.7-fold in MM cells co-cultured with normal, overweight, or obese adipocytes, respectively compared to MM cells cultured alone. In regard to caspase-3, bortezomib or triple-drug treatment converted all the full-length (inactive) caspase-3 to cleaved caspase-3 (Figure 1C) in non-co-cultured RPMI 8226 MM cells. However, all MM cells co-cultured with normal, overweight, or obese adipocytes only had partial activation of caspase-3, with 48–50% (normal or obese adipocytes), or 61% (overweight adipocytes) of inactive caspase-3 being cleaved/activated within 24 h.

### 3.2. Adipocytes from Overweight and Obese Individuals Protect MM Cells from Drug-Induced Reductions in Survival Factors

**Figure 2:**
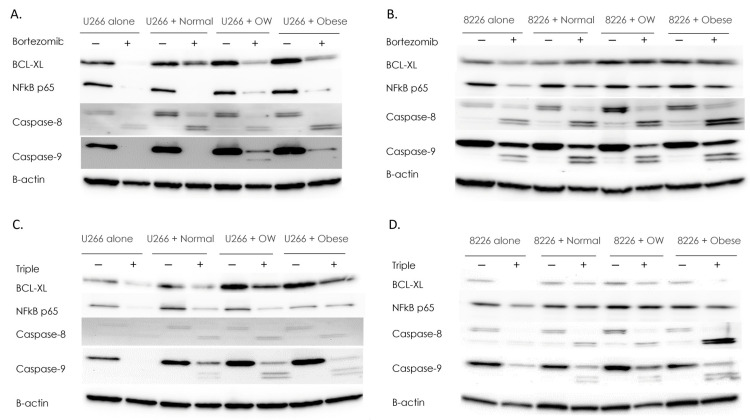
Adipocytes protect MM cells from drug-induced reductions in survival factors. Western analysis for survival factors in U266B1 (**A**) or RPMI 8226 (**B**) MM cells +/− bortezomib. Western analysis for survival factors in U266B1 (**C**) or RPMI 8226 (**D**) MM cells +/− bortezomib, lenalidomide, and dexamethasone in combination (“triple”). Control or drug-treated MM cells were cultured alone or in the presence of adipocytes from normal, overweight, or obese patients. All Westerns were performed a minimum of three times. Densitometry was performed using NIH Image J and bands were normalized to B-actin which served as a housekeeping gene. Uncropped WB images are shown in Figure S2.

In MM, overexpression of factors such as NFκB p65 and BCL-xL contribute to heightened survival, proliferation, and resistance to anti-cancer therapies, and contributes to CAM-DR [40,41]. To test whether obesity affected the expression of these factors in response to bortezomib, Western blotting was performed. In non-co-cultured U266B1 MM cells, the addition of bortezomib completely inhibited expression of NFκB (Figure 2A). When MM cells were co-cultured with adipocytes from overweight or obese individuals, NFκB only had partial inhibition, yielding levels eight-fold higher than non-co-cultured cells. Similarly, BCL-xL expression was completely inhibited in non-co-cultured U266B1 cells treated with bortezomib. However, when U266B1 MM cells were co-cultured with overweight or obese adipocytes, bortezomib had a more attenuated effect in decreasing BCL-xL, reducing expression by 50%.

In RPMI 8226 MM cells, co-culture with adipocytes from overweight or obese individuals increased the survival proteins NFκB p65 (1.6–2.1-fold) and BCL-xL (2 to 2.6-fold), compared to non-co-cultured cells (Figure 2B). While bortezomib inhibited NFκB p65 and BCL-xL 40% in RPMI 8226 MM cells cultured alone, MM cells co-cultured with overweight or obese adipocytes and treated with bortezomib displayed no reduction in these survival factors.

Caspases are a family of proteases that can mediate the anti-drug effects of bortezomib and lenalidomide in MM. However, the overactivation of caspase-8 has been found in drug-resistant myeloma cells and, in some cancers, caspases such as caspase-8 and caspase-9 are linked to increased tumorigenesis, survival and invasion [42,43,44]. Therefore, we assessed whether U266B1 and 8226 cells co-cultured with adipocytes and treated with bortezomib had altered caspase-8 and caspase-9 levels. As expected, bortezomib treatment of RPMI 8226 or U266B1 cells converted full-length caspase-8 to cleaved (activated) caspase-8 (Figure 2A,B). U266B1 cells co-cultured with normal, overweight, or obese adipocytes increased activation of caspase-8 in response to bortezomib 2.3-fold (normal), 1.2-fold (overweight), and 2.5-fold (obese) higher than non-co-cultured MM cells. RPMI 8226 cells showed a similar pattern, increasing activation of caspase-8 2-fold, 3-fold, and 2.5-fold when co-cultured with normal, overweight, or obese adipocytes compared to non-co-cultured MM cells treated with bortezomib. Caspase-9 exhibited similar patterns to caspase-8 (Figure 2B). In U266B1 cells, activated caspase-9 was only seen in bortezomib-treated cells co-cultured with overweight and obese adipocytes. RPMI 8226 cells cultured with overweight or obese adipocytes had two-fold higher activation of cleaved caspase-9 compared to non-co-cultured cells.

MM patients are typically treated with a combination of drugs that have different but overlapping anti-MM properties, as this can be more effective than treatment with single agents. To test whether a triple treatment of bortezomib, lenalidomide, and dexamethasone was more effective than bortezomib alone and unaffected by the presence of adipocytes, Western analysis was performed. Similar to when bortezomib was added as a single agent, BCL-xL was inhibited completely (RPMI 8226) or inhibited 63% (U266B1) in MM cells cultured alone and treated with a triple-drug cocktail (Figure 2C,D). NFκB p65 was inhibited 44% in RPMI 8226 and 95% in U266B1 cells when no adipocytes were present. Co-culture with adipocytes restored NFκB p65 protein levels to those of control. Triple-drug treatment was 50% less effective in reducing NFκB in U266B1 co-cultured cells and displayed no reduction in NFκB in RPMI-8226 cells.

As expected, triple-drug treatment of RPMI 8226 or U266B1 cells converted full length caspase-8 to cleaved (activated) caspase-8, although only a weak signal was detected (Figure 2C,D). However, U266B1 cells co-cultured with normal, overweight, or obese adipocytes and treated with triple-drug cocktails increased activated caspase-8 18-fold (normal), 32-fold (overweight), and 20-fold (obese) higher than non-co-cultured MM cells. RPMI 8226 cells showed a similar pattern, increasing activation of caspase-8 1.9-fold, 2-fold, and 20.3-fold when co-cultured with normal, overweight, or obese adipocytes, respectively, compared to non-co-cultured MM cells treated with triple-drug cocktails. Caspase-9 exhibited similar patterns to caspase-8 (Figure 2C,D), with treated co-cultured U266B1 cells increasing caspase-9 cleavage 6-fold, 29-fold, and 19-fold (normal, overweight, or obese adipocytes, respectively), compared to treated non-co-cultured MM cells, and treated co-cultured RPMI 8226 cells increasing caspase-9 cleavage 6.4-fold, 5.5-fold, and 10.2-fold in (normal, overweight, or obese, respectively), compared to treated non-co-cultured MM cells.

### 3.3. Adipocytes Prevent Drug-Induced Reductions in P-Glycoprotein and MRP1 Transporter Levels in MM Cells

**Figure 3:**
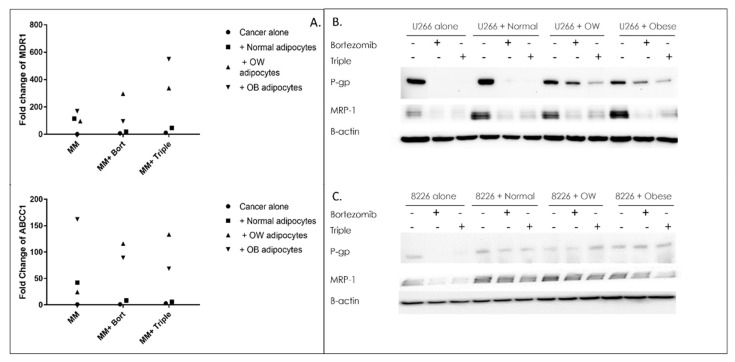
Adipocytes block drug-induced reductions in efflux pumps in MM cells. qPCR of MDR1 or ABCC1 genes (**A**), or Western analysis (**B**,**C**) of their associated proteins P-glycoprotein, and MRP1 in U266B1 (**B**) or RPMI 8226 (**C**) MM cells +/− bortezomib, or bortezomib, lenalidomide, and dexamethasone in combination (“triple”). Control or drug-treated MM cells were cultured alone or in the presence of adipocytes from normal, overweight, or obese patients 24 h prior to RNA or protein isolation. All qPCRs or Western blots were normalized to GAPDH (qPCR) or B-actin (Western blotting), which served as a housekeeping gene. Uncropped WB images are shown in Figure S3.

One of the most important mechanisms underlying cancer drug resistance is the activation of ATP-dependent efflux pumps for chemotherapeutic agents. To see whether P-glycoprotein and MRP1 pumps, or their associated genes, MDR1 and ABCC1, were activated in drug-treated MM cells in response to adipocytes, qPCR and Western analysis were performed. MDR1 gene expression was increased 7.3 and 11.1-fold in non-co-cultured MM cells treated with bortezomib or triple-drug treatment compared to MM cells cultured without drugs. ABCC1 gene expression was increased 1.3 and 2.8-fold in non-co-cultured MM cells treated with bortezomib or triple-drug treatment, compared to MM cells cultured without drugs (Figure 3A). However, just the presence of adipocytes alone, irrespective of drug treatment, significantly elevated both MDR1 and ABCC1 genes (Figure 3A), achieving levels 95–168-fold (MDR1) or 24–162 (ABCC1) higher than controls. When MM cells were co-cultured with overweight or obese adipocytes and treated with bortezomib, gene transcription of MDR1 was significantly (42-fold for overweight, 13-fold for obese) elevated compared to bortezomib-treated MM cells cultured alone. Similarly, co-culture with overweight or obese adipocytes and treatment with bortezomib increased ABCC1 gene expression 90-fold (overweight) or 70-fold (obese) compared to non-co-cultured MM cells treated with bortezomib. Similar patterns were seen in MM cells co-cultured with overweight or obese adipocytes and treated with triple drugs, elevating ABCC1 gene expression 24-fold (obese) or 47-fold (OW) and increasing MDR1 gene expression 31-fold (OW) and 49-fold (obese) compared to non-co-cultured MM cells treated with triple drugs.

Western analysis also revealed an upregulation in drug-resistance proteins in co-cultured MM cells. In U266B1 and RPMI 8226 MM cells treated with bortezomib or triple treatment, P-gp protein expression was completely inhibited compared to untreated controls (Figure 3B,C). However, in U266B1 cells cultured with overweight or obese adipocytes and treated with bortezomib, P-gp was inhibited 35% and 52%, respectively (Figure 3B). Cells cultured with overweight or obese adipocytes and treated with a triple-drug regimen decreased P-gp protein levels 77% and 73%, respectively. In RPMI 8226 cells, there was no inhibition of P-gp in cells cultured with overweight or obese adipocytes and treated with bortezomib or triple-drug cocktail (Figure 3C), compared to untreated controls. MRP1, another efflux pump often activated in cancer, followed a similar pattern to P-gp. Treatment of non-co-cultured U266B1 or RPMI 8226 cells with bortezomib or triple-drug combinations resulted in near total inhibition in MRP1. However, in U266B1 cells, co-culturing with normal, overweight, or obese adipocytes (without drugs) increased MRP1 5.5-fold, 3.8-fold, and 4.9-fold respectively compared to non-co-cultured control cells, and drug treated co-cultured cells had MRP1 50% higher than drug treated non-co-cultured cells. In RPMI 8226 cells, co-culturing cells with adipocytes alone (no drugs) had two-fold higher levels of MRP1. There was little to no reduction in co-cultured cells upon treatment with bortezomib or with the bortezomib, lenalidomide, and dexamethasone combination.

### 3.4. MM Cells Co-Cultured with Adipocytes from Overweight or Obese Individuals Develop an Increased Endothelial Cell Tube Network and Are More Resistant to the Effects of Drugs

**Figure 4:**
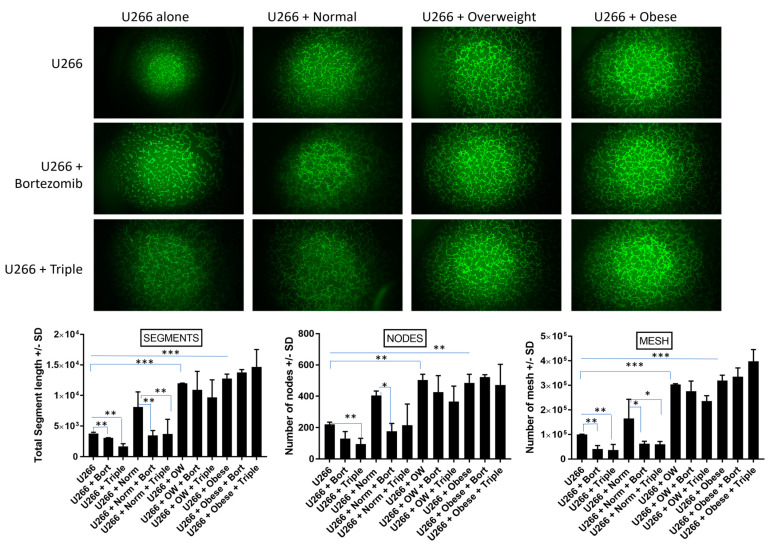
MM cells co-cultured with adipocytes from overweight or obese individuals display increased in vitro angiogenesis and are more resistant to drug-induced reductions in endothelial cell tube network. 3B11 mouse endothelial cells were serum starved. In total, 10^5^ cells/well were subjected to conditioned media from MM cells grown alone and treated with bortezomib, or bortezomib, lenalidomide, and dexamethasone in combination (“triple”), or cultured in the presence of adipocytes from normal, overweight, or obese patients. Tube networks were grown for 6 h and imaged under 4× magnification with a fluorescence microscope. The numbers of nodes, mesh, and segments were quantified using NIH Image J with Angiogenesis Plugin as described elsewhere [40]. Significance values of *p* < 0.05 are indicated in figures with a single asterisk *, *p* < 0.01 with a double asterisk **, and *p* < 0.001 with a triple asterisk ***.

To assess whether MM cells grown in the presence of adipocytes were more resistant to the anti-angiogenic effects of bortezomib or triple-drug cocktail, an endothelial tube formation assay was performed [38,39]. 3B11 endothelial cells subjected to conditioned media from U266B1 MM cells co-cultured with adipocytes from normal, overweight, or obese individuals developed 2.1 (ns), 3.2 (*p* < 0.001), and 3.4 (*p* < 0.001) times the number of segments, 1.8 (ns), 2.2 (*p* < 0.01), 2.2 (*p* < 0.01) times the number of nodes, and 1.6 (ns), 3 (*p* < 0.001), and 3.2 (*p* < 0.001) times the number of mesh, respectively, than MM cells cultured alone (Figure 4). Additionally, while treatment with bortezomib alone or bortezomib, lenalidomide, and dexamethasone triple treatment significantly (*p* < 0.01) reduced endothelial tube formation in MM cells cultured alone or co-cultured with adipocytes from normal weight individuals compared to non-drug treated cells, treatment with single or triple-drug cocktail was unable to decrease endothelial tube formation in cells co-cultured with adipocytes from overweight or obese patients.

### 3.5. MMP-2 Enzymatic Activity Is Significantly Higher in MM Cells Co-Cultured with Adipocytes

**Figure 5:**
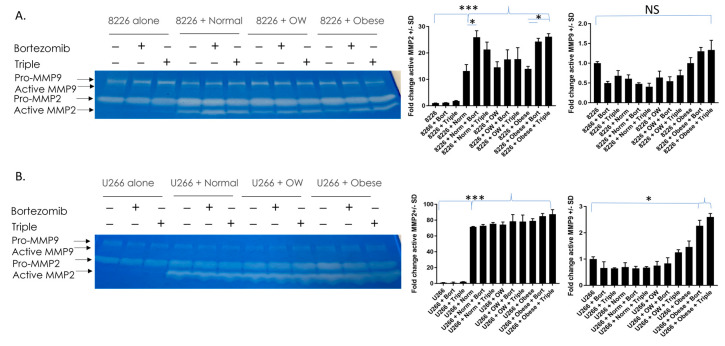
MMP-2 enzymatic activity is significantly higher in MM cells co-cultured with adipocytes. MMP-2 and MMP-9 activity were measured in RPMI 8226 (**A**) and U266B1 (**B**) MM cells. MMP-2 is significantly elevated in cells co-cultured with adipocytes. MMP-9 activity was elevated in U266B1 cells co-cultured with adipocytes from obese patients and treated with bortezomib or triple treatment. * *p* ≤ 0.05. *** *p* ≤ 0.001. Uncropped zymogram gels are shown in Figure S5.

Patients with MM have heightened MMP-2, a factor that contributes to cancer invasion and angiogenesis [45,46,47]. To see if MMP-2 activity was inhibited when co-cultured and non-co-cultured MM cells were treated with bortezomib or bortezomib/lenalidomide/dexamethasone triple treatment, zymography was performed. The levels of active MMP-2 in RPMI-8226 cells were barely detectable in MM cells cultured alone +/− drugs (Figure 5A). However, the presence of adipocytes from normal, overweight, or obese individuals alone increased the activity of MMP-2 in cancer cells (*p* < 0.0001). Additionally, in RPMI-8226 cells, MMP-2 activity was significantly (*p* < 0.01) higher in normal co-cultured cells treated with bortezomib or obese co-cultured cells treated with bortezomib or triple-drug cocktails compared to untreated co-cultured cells. In U266B1 cells, the levels of active MMP-2 were barely detectable in cells cultured alone +/− drugs (Figure 5B). Like RPMI-8226 cells, the presence of adipocytes from normal, overweight, or obese individuals alone increased the activity of MMP-2 in cancer cells (*p* < 0.0001). We also measured the activity of MMP-9, another factor important to cancer progression and angiogenesis. Unlike MMP-2, the majority of MMP-9 remained in the pro-form. While no differences in active MMP-9 were observed in RPMI-8226 cells, in U266B1 cells active MMP-9 (lower band in the doublet) was two-fold higher in drug-treated MM cells co-cultured with adipocytes from obese patients compared to non-co-cultured drug-treated MM cells.

## 4. Discussion

It is widely recognized that signals from the bone marrow microenvironment can alter the migration, proliferation, and survival of MM cells, and confer drug resistance [48]. Obesity is a risk factor for MM, contributing to both an increased risk of MM development and poor survival outcomes [6,7,8,9]. In response to obesity, adipocytes, the predominant cell type of yellow bone marrow, can enlarge, expand, and alter their cytokine and hormone profiles. Since MM cells reside in the bone marrow and interact with neighboring adipocytes, any changes in adipocyte populations surrounding the MM cells can have a profound effect on cancer growth or invasiveness.

We have previously reported that signals from adipocytes increase MM cell growth, adhesion, and angiogenesis, with more pronounced effects when adipocytes came from overweight or obese individuals [18]. Several different mechanisms by our lab and others have been suggested for how adipocytes support the MM pathological response [18,19,49,50]. Dysfunctional adipocytes display alterations in metabolic, endocrine, and inflammatory signaling, resulting in impaired glucose and lipid metabolism, insulin resistance, and oxidative stress [18,51]. Adipocytes in bone marrow can serve as a fuel source and provide free fatty acids to MM cells, supporting their growth and survival [49]. Adipocytes can also protect MM cells from chemotherapy-induced apoptosis through activation of autophagy or induction of m^6^A methylation in exosomal long non-coding RNAs [52,53]. In this report, we found that adipocytes could enhance MM drug resistance, decreasing the effectiveness of bortezomib or a triple-drug treatment of bortezomib, lenalidomide, and dexamethasone. We further found that the ability of adipocytes to enhance drug resistance mechanisms was often more pronounced when adipocytes came from patients with higher BMIs.

Multiple intrinsic and extrinsic mechanisms contribute to MM drug resistance and subsequent therapeutic failure [54]. Among them, activation of the ATP-dependent efflux pumps P-glycoprotein/MDR, and their related factor MRP1, has considerable pharmacological importance, as they decrease the efficacy of chemotherapeutic agents by reducing the intracellular concentration of drugs [30]. In this report, we showed that, while bortezomib or triple-drug treatment was very effective in completely inhibiting P-glycoprotein or MRP1 protein levels in MM cells cultured alone, drugs were unable to fully inhibit transporters in MM cells co-cultured with overweight or obese adipocytes. This is relevant in that relapsed MM patients have increased P-glycoprotein levels when compared to non-treated MM patients, and MM-resistant cells have increased P-glycoprotein or expression of its gene MDR1 [35,36,37]. Both MDR1 and its protein P-glycoprotein can be induced by STAT3, a factor we have previously shown was significantly elevated in MM cells co-cultured with overweight or obese adipocytes [18,55,56]. This induction can contribute to resistance to chemotherapeutics.

Another important mechanism for chemoresistance in MM cells is CAM-DR [57]. In CAM-DR, cell adhesion between MM cells and cells of the tumor microenvironment can trigger activation of NFκB-regulated survival pathways and prevent drug-induced apoptosis [32,58]. We have previously shown that adipocytes cause MM cell adhesion in a manner that is positively correlated with BMI [18]. Further, we have shown that co-culture of adipocytes with MM cells causes upregulation in alpha 4 integrin, a factor important for CAM-DR [18]. Here, we evaluated the ability of bortezomib or combination drug treatments to decrease the expression of survival factors in MM cells cultured alone or cultured in the presence of adipocytes. As expected, bortezomib or triple-drug treatment strongly inhibited NFκB and BCL-xL expression in MM cells cultured alone. However, survival factors were only partially suppressed in drug-treated co-cultured cells, suggesting that adipocytes help MM cells become resistant to the effects of anti-cancer drugs. Both NFκB and BCL-xL are strongly correlated with pathogenesis of MM [41,59]. Activation of NFκB signaling is found in the large majority of MM patients, and inhibition of NFκB can decrease MM proliferation and promote apoptosis [40,59]. In a similar fashion, BCL-xL is higher in MM patients and correlates with resistance to treatment [41,60]. The ability of adipocytes to maintain survival signals in drug-treated MM cells correlated with our viability data, which showed partial resistance to drug-induced reductions in cell viability in co-cultured cells (Figure 1B).

Historically, caspases have been associated with the initiation of apoptosis [61]. Our immune system and many therapeutic agents engage this pathway as a means of effectively eliminating cancerous cells. However, a secondary pro-tumorigenic role for caspases has recently been established, as caspase activation can increase cancer cell proliferation, invasion, and angiogenesis [62]. Typically, bortezomib and lenalidomide activate caspase-8 and caspase-9, inducing apoptosis [63,64]. However, recent reports indicate that in certain cancers these caspases can have paradoxical functions, serving a pro-tumorigenic role [65,66]. In this manner, high levels of caspase-8 are found in lenalidomide-resistant RPMI-8226 MM cells, and activation of caspase-8 weakens the anti-myeloma effect of bortezomib and lenalidomide [66] In glioblastoma, caspase-8 activates NFκB signaling, leading to enhanced proliferation, angiogenesis, and chemoresistance [65]. Caspase-9b, one of the cleaved isoforms of caspase-9, can also activate the NFκB pathway, increase tumorigenesis, and cause chemoresistance [44]. In this report, we found significantly higher levels of cleaved caspase-8 and caspase-9b in drug-treated co-cultured cells. Thus, activation of caspase-8 and caspase-9 may be partially responsible for the heightened NFκB signaling found in MM cells co-cultured with adipocytes. Additionally, full length caspase-8 was also heightened in MM cells co-cultured with adipocytes compared to MM cells cultured alone. This aligns with a previous report which showed that caspase-8 expression is increased in adipocytes from humans with obesity and diabetes and blockade of caspase-8 prevented weight gain in mice [67]. Thus, it may play a role in regulating whole body energy.

It is well known that an angiogenic switch can occur in MM, with heightened angiogenesis leading to cancer progression [68]. To assess whether MM cells cultured with adipocytes were less responsive to drug-induced reductions in angiogenesis, an endothelial tube formation assay was performed [38,39]. Bortezomib or triple-drug treatment significantly decreased endothelial tube formation in non-co-cultured MM cells or those co-cultured with adipocytes from patients with normal BMIs. However, endothelial cells were more resistant to drug-induced decreases in angiogenesis when subjected to signals from MM cells co-cultured with adipocytes from overweight or obese patients. A variety of factors can cause angiogenesis. Among them, the ability of MMP-2 to stimulate angiogenesis has been known for decades [46,47]. In drug-treated RPMI 8226 and U266B1 MM cells, we found nearly undetectable levels of active MMP-2 when cells were cultured alone. In both cell lines, MMP-2 activity was drastically elevated when MM cells were co-cultured with adipocytes. Similar to our findings, others have shown that bone marrow stromal cells can produce MMP-2, which in turn promotes NFκB activity in MM cells [69]. Additionally, in a prior publication, we showed that adipocytes from overweight or obese patients secreted significantly higher levels of IL-6 [18]. IL-6 is a direct activator of STAT3, which we have shown is substantially elevated in MM cells co-cultured with adipocytes from patients with higher BMIs [18]. STAT3 activation directly regulates the expression of MMP2, suggesting this factor may play a crucial role in MMP2-associated invasion or angiogenesis [70].

One limitation of this study was the lack of diversity in ASCs obtained from patients. Most ASCs used in this study were from white females, as they accounted for the preponderance of patients undergoing abdominal liposuctions from which the ASCs were obtained. Future directions will include expanding the repertoire of samples to have an increased representation of males and underrepresented populations to better reflect the MM patient population.

**Figure 6:**
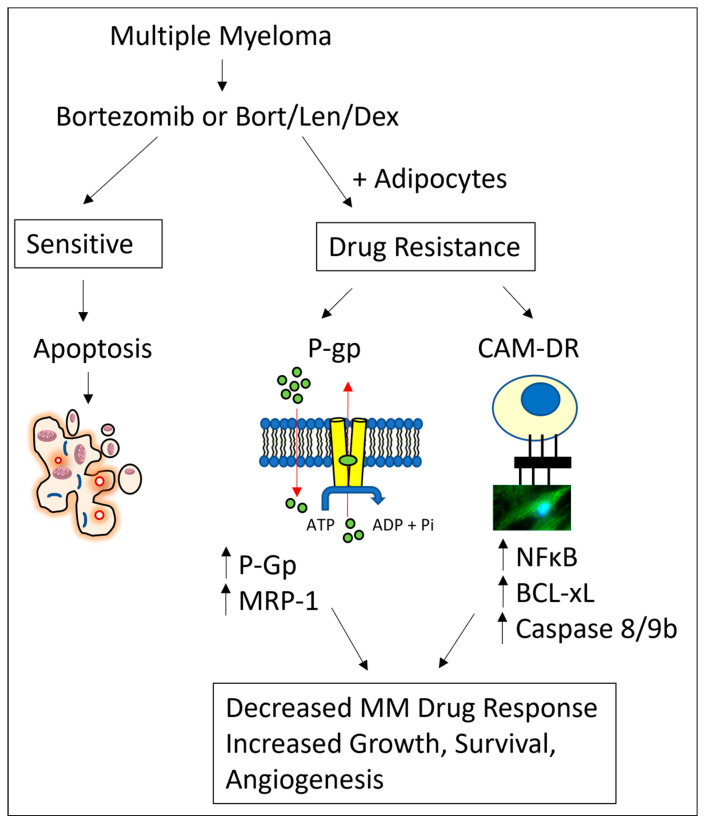
Schematic of adipocyte-mediated drug resistance in MM cells. Bortezomib alone or bortezomib/lenalidomide/dexamethasone combination treatment decreases MM cell growth, survival, and angiogenesis. MM cells cultured in the presence of adipocytes have increased MDR and CAM-DR drug resistance pathways, leading to increases in MM cell growth, survival and angiogenesis.

In summary, we suggest that adipocytes drive P-glycoprotein and CAM-DR mediated drug resistance in MM (Figure 6), with an augmented effect when MM cells were cultured with adipocytes from higher BMI categories. Understanding the interactions between MM cells and cells that comprise the bone marrow niche is important for developing new therapies that can target the bone marrow microenvironment and improve MM patient outcomes.

## Figures and Tables

**Table 1:** Patient Information.

ID Number	BMI (kg/m^2^)	Location	Gender	Age
L030702T	18	Thigh/Abdomen	Female	26
L053001	20.45	Abdomen	Female	28
L092815A	21	Thigh/Abdomen	Female	35
ASC100614B	21.5	Abdomen	Female	34
ASC100610B	23.3	Abdomen	Female	40
LM060710C	24.5	Abdomen	Female	67
L012502	25.3	Abdomen	Male	40
L120116E	26.5	Abdomen	Female	52
ASC102014A	26.2	Abdomen	Female	48
SL0064	27.3	Abdomen/Thigh/Neck/Flanks	Female	50
SL0071	27.5	Abdomen/Thigh/Hip/Flank	Female	49
SL0065	27.6	Thigh/Flanks/Hips/Abdomen	Female	44
L071212	30.6	Abdomen	Female	35
L080218B	31.9	Abdomen	Female	39
L062016A	32.8	Abdomen	Female	45
ASC050709B	34.8	Abdomen/Hip	Female	42
L051319B	35.4	Abdomen/Thigh/Hip	Female	56
L072709	38	Abdomen/Hip	Female	39
L050718A	44.5	Abdomen	Male	51

**Table 2:** Primer Sequences.

Oligo Name	Sequence (5′ to 3′)
Human ABCB1 Forward	TGCTCAGACAGGATGTGAGTTG
Human ABCB1 Reverse	TTACAGCAAGCCTGGAACCTA
Human ABCC1 Forward	TCTACCTCCTGTGGCTGAATCTG
Human ABCC1 Reverse	CACCTGATACGTCTTGGTCTTCAT
Human GADDH Forward	AAGGTGAAGGTCGGAGTCAA
Human GAPDH Reverse	AATGAAGGGGTCATTGATGG

## Data Availability

Data for this study are contained in a public repository (https://osf.io/d4bmr/, accessed on 12 July 2023).

## Supplementary Materials

The following supporting information can be downloaded at: https://www.mdpi.com/article/10.3390/cancers15174347/s1, Figures S1, S2 and S3: uncropped Western blot images for Figure 1, Figure 2 and Figure 3, respectively. Figure S4: Densitometry graphs for Western Blot images. Figure S5: Uncropped Zymogram gels.
