# Supplementary material for: Unlocking Drug Resistance in Multiple Myeloma: Adipocytes as Modulators of Treatment Response
Source: Cancers (Basel). 2023 Aug 31;15(17):4347. doi: 10.3390/cancers15174347 (PMC10486466; doi:10.3390/cancers15174347)
Supplement: Supplementary file 1 [file cancers-15-04347-s001.zip › cancers-2527961-supplementary.pdf]

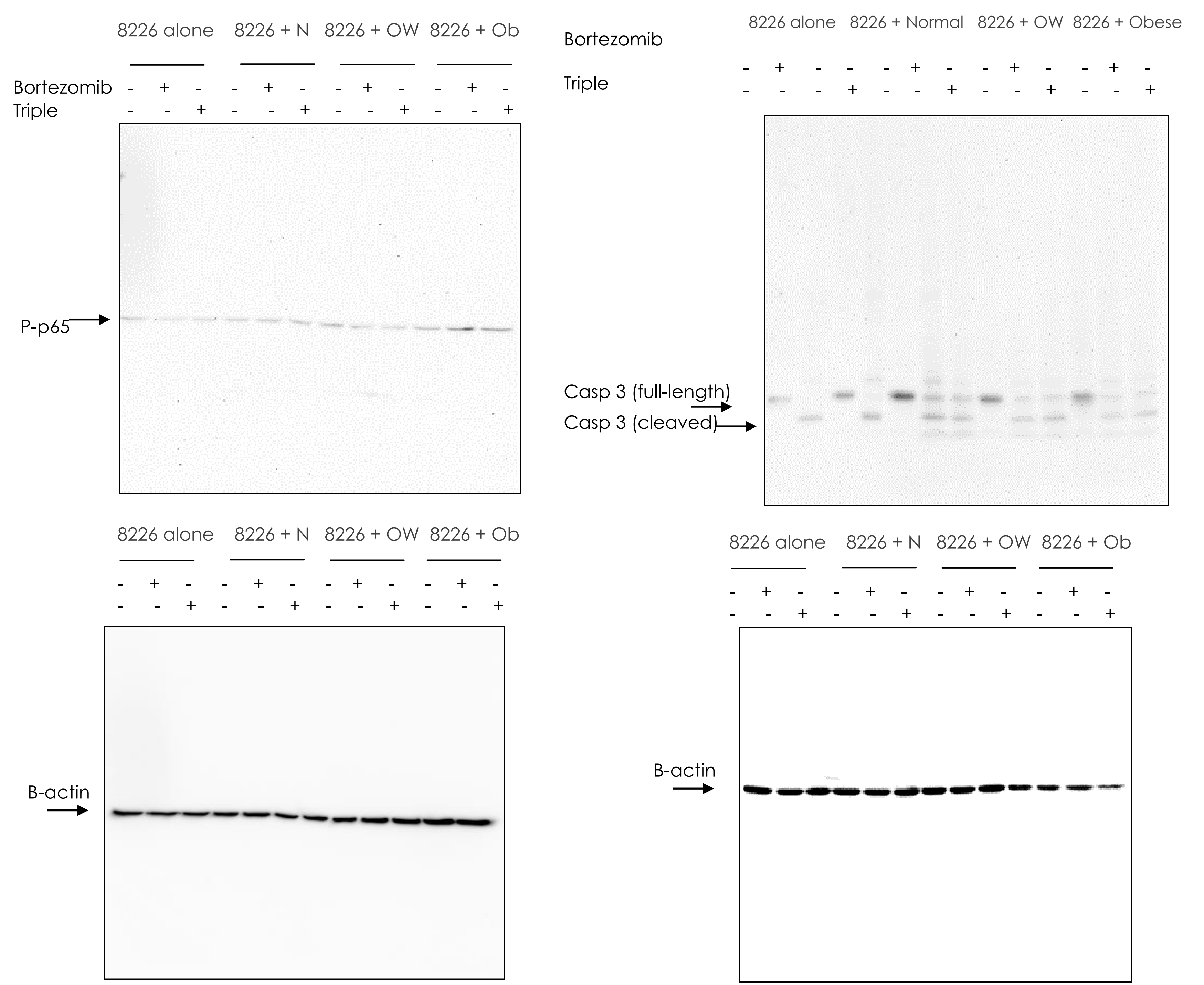

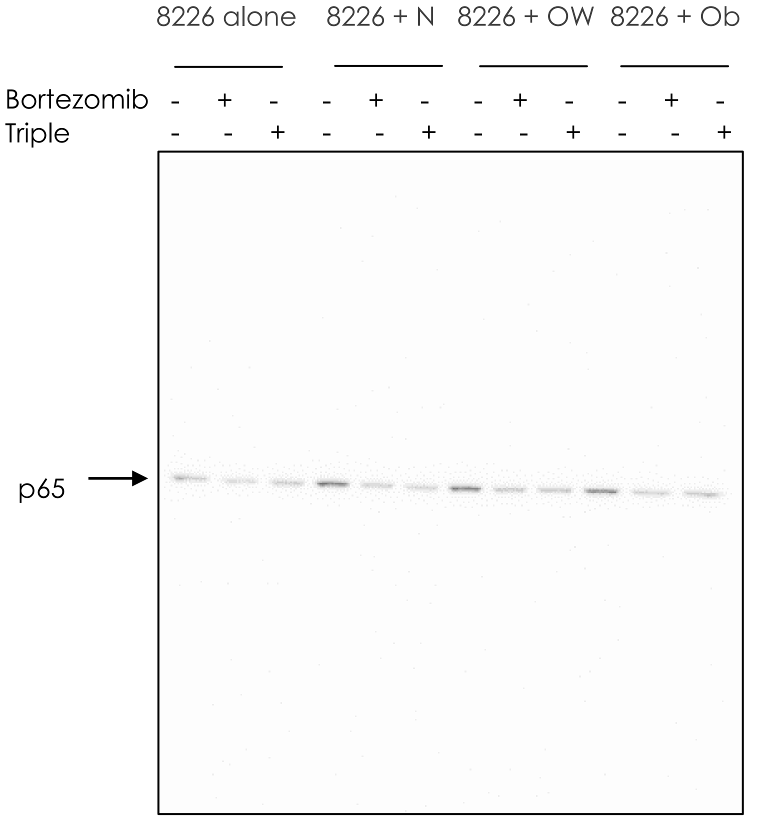

Figure S1. Uncropped WB images for Figure 1.

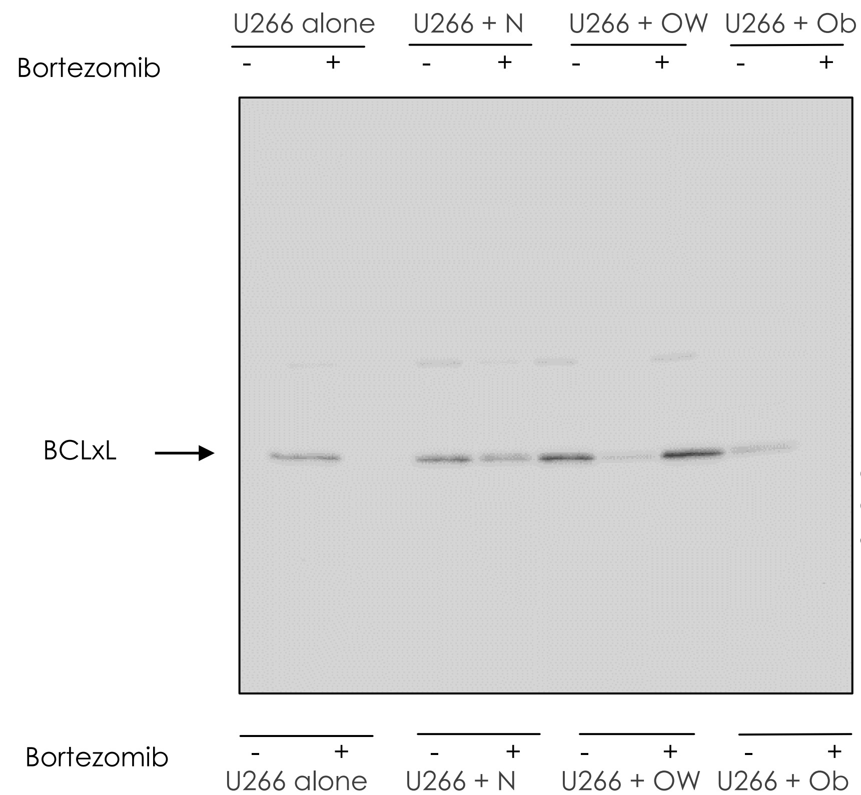

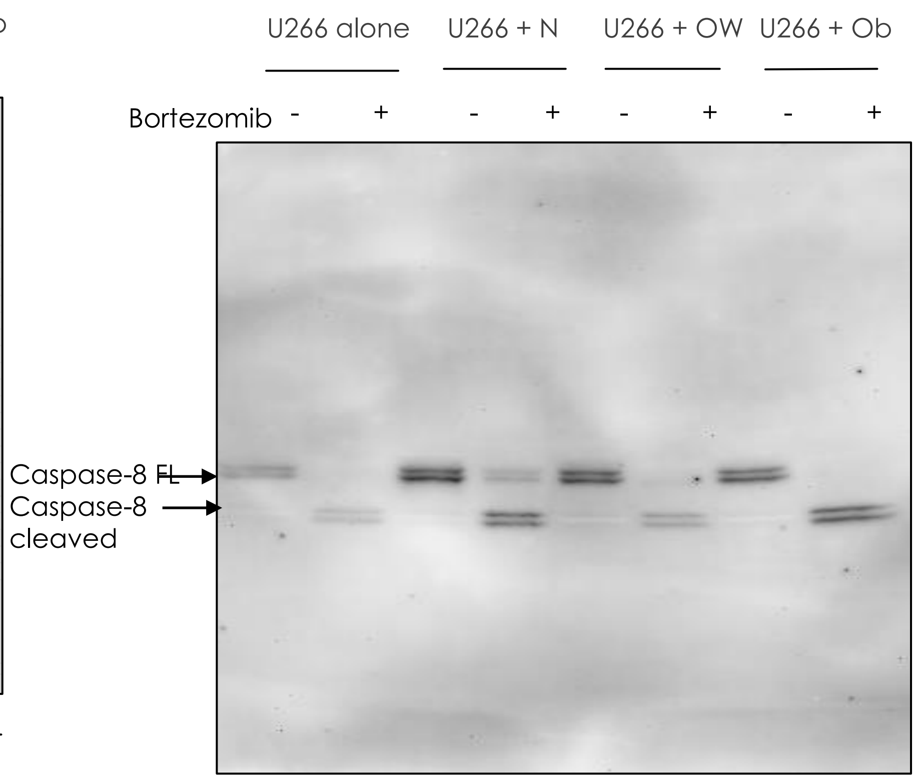

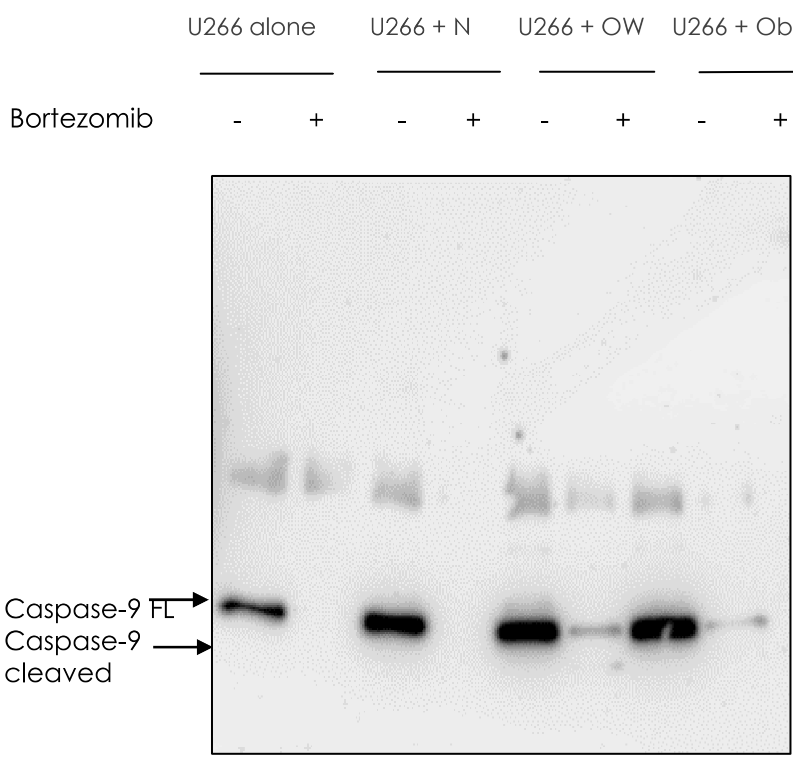

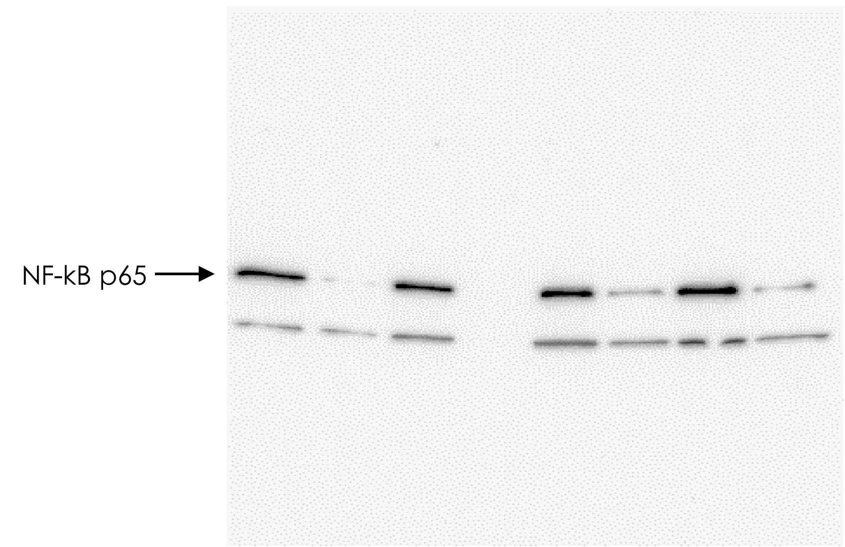

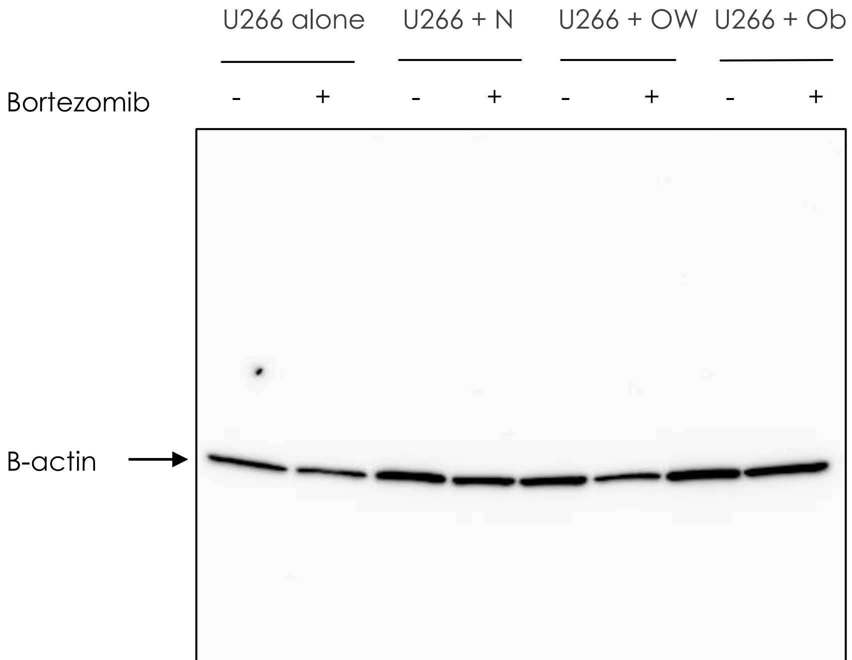

Figure S2. Uncropped WB images for Figure 2

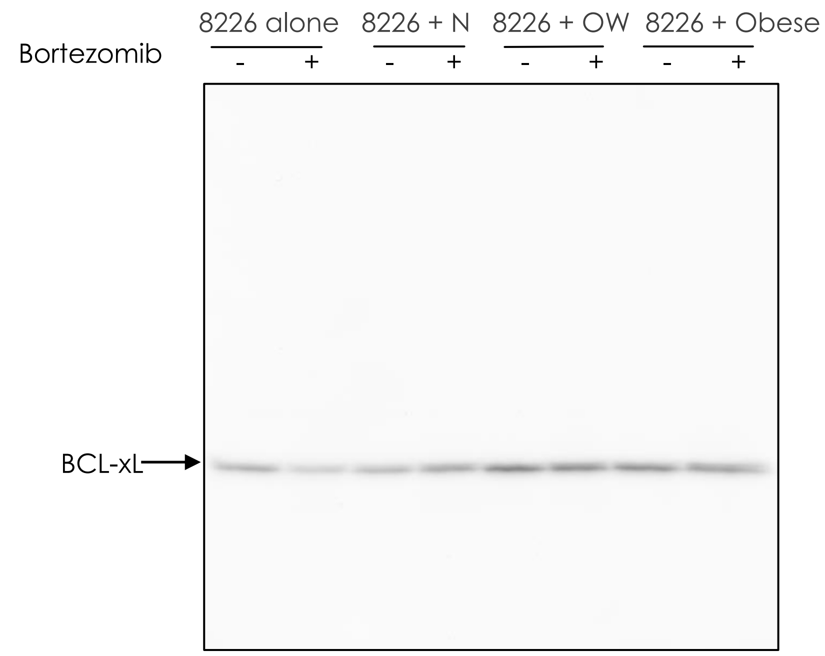

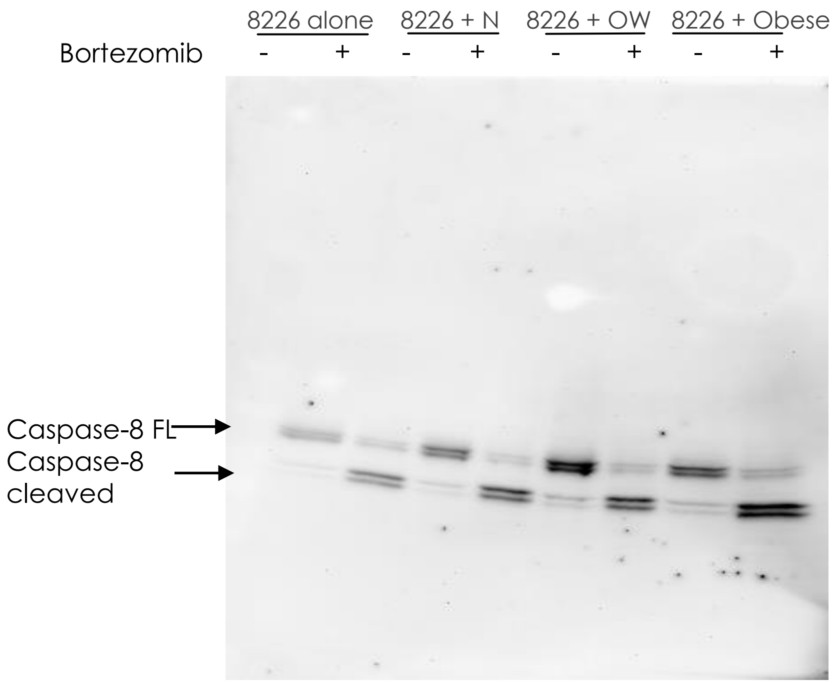

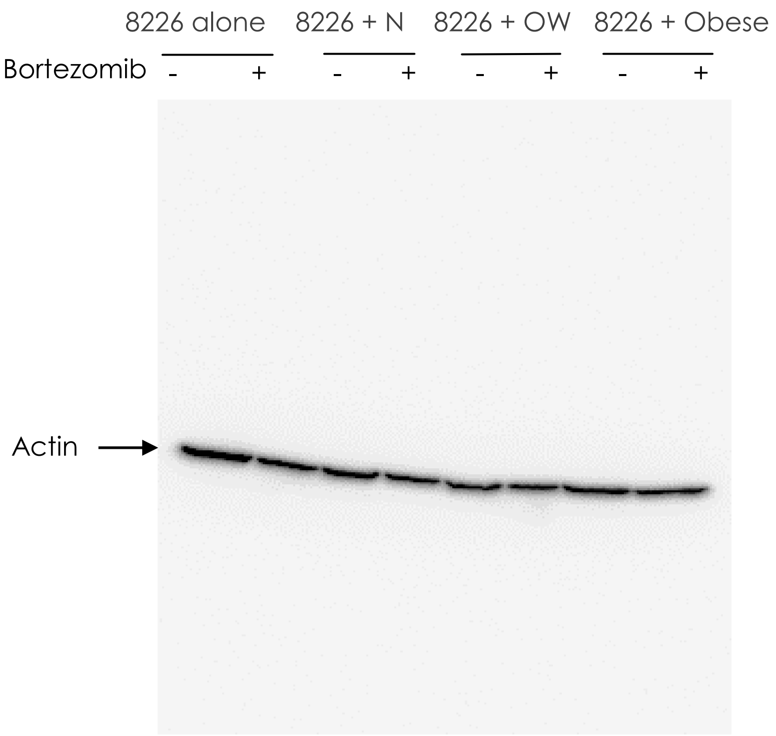

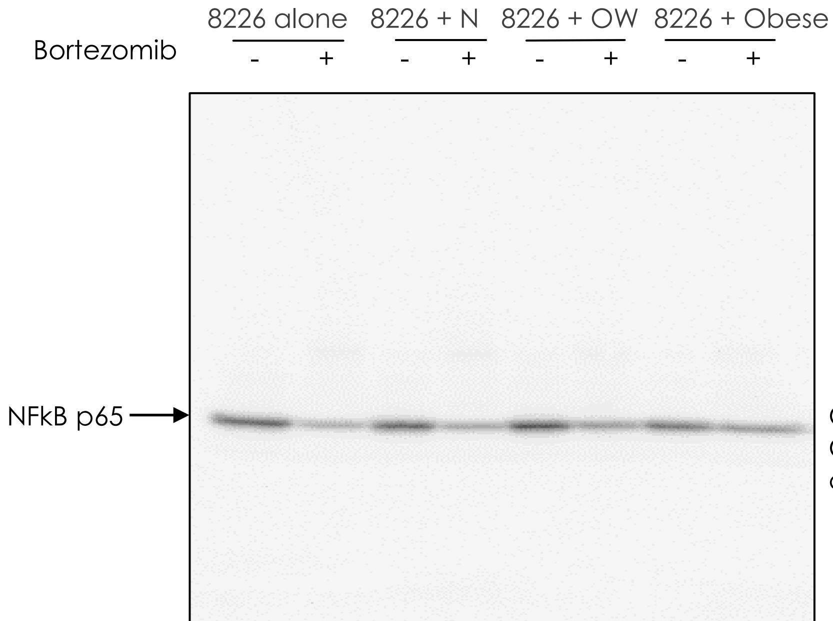

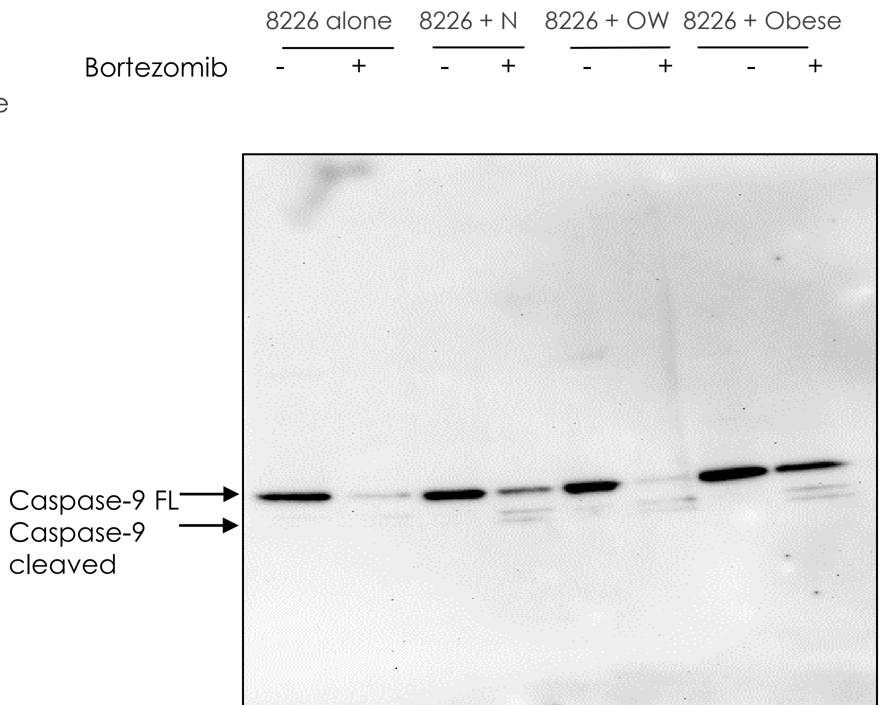

Figure S2. Uncropped WB images for Figure 2

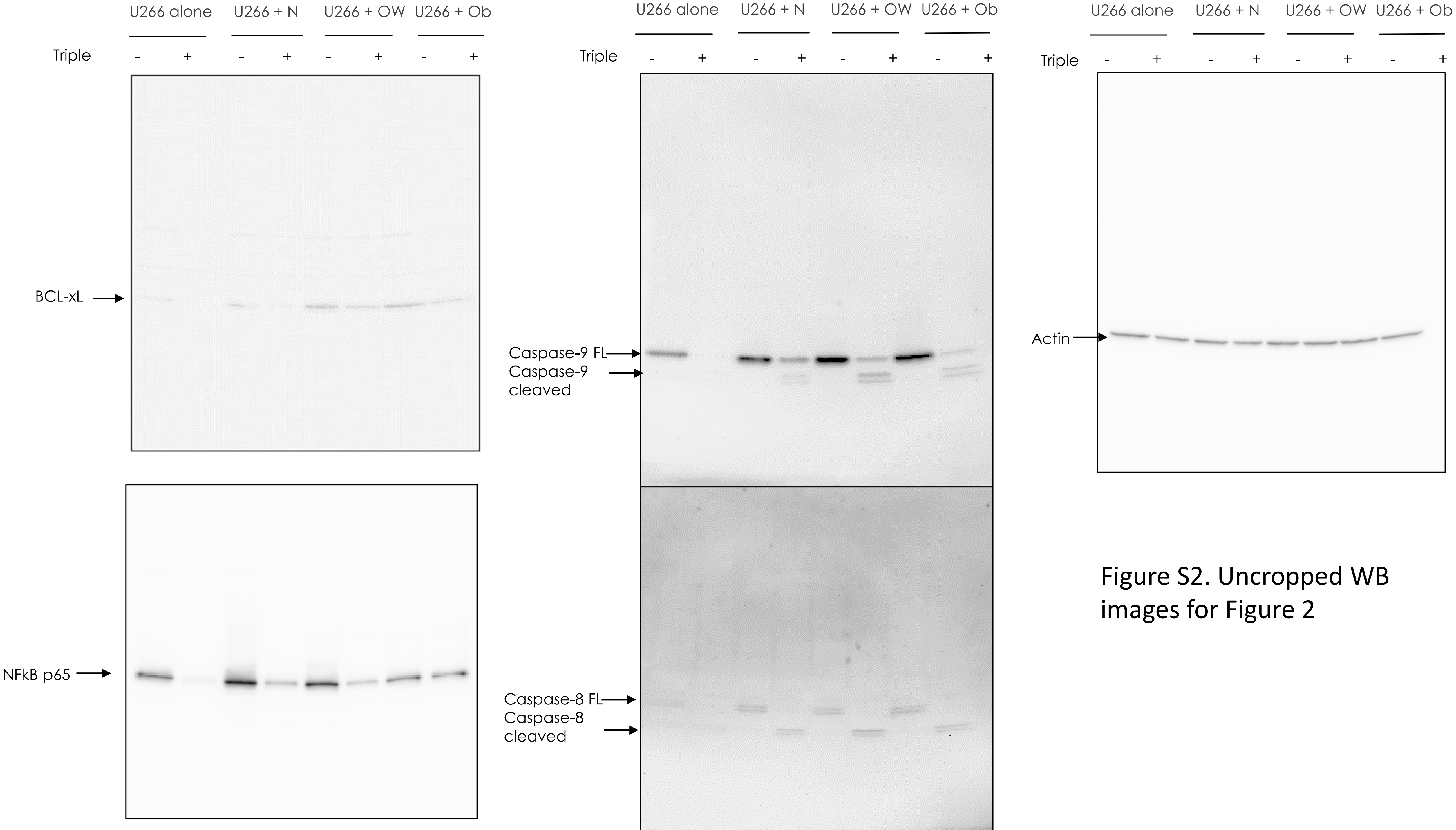

Figure S2. Uncropped WB images for Figure 2

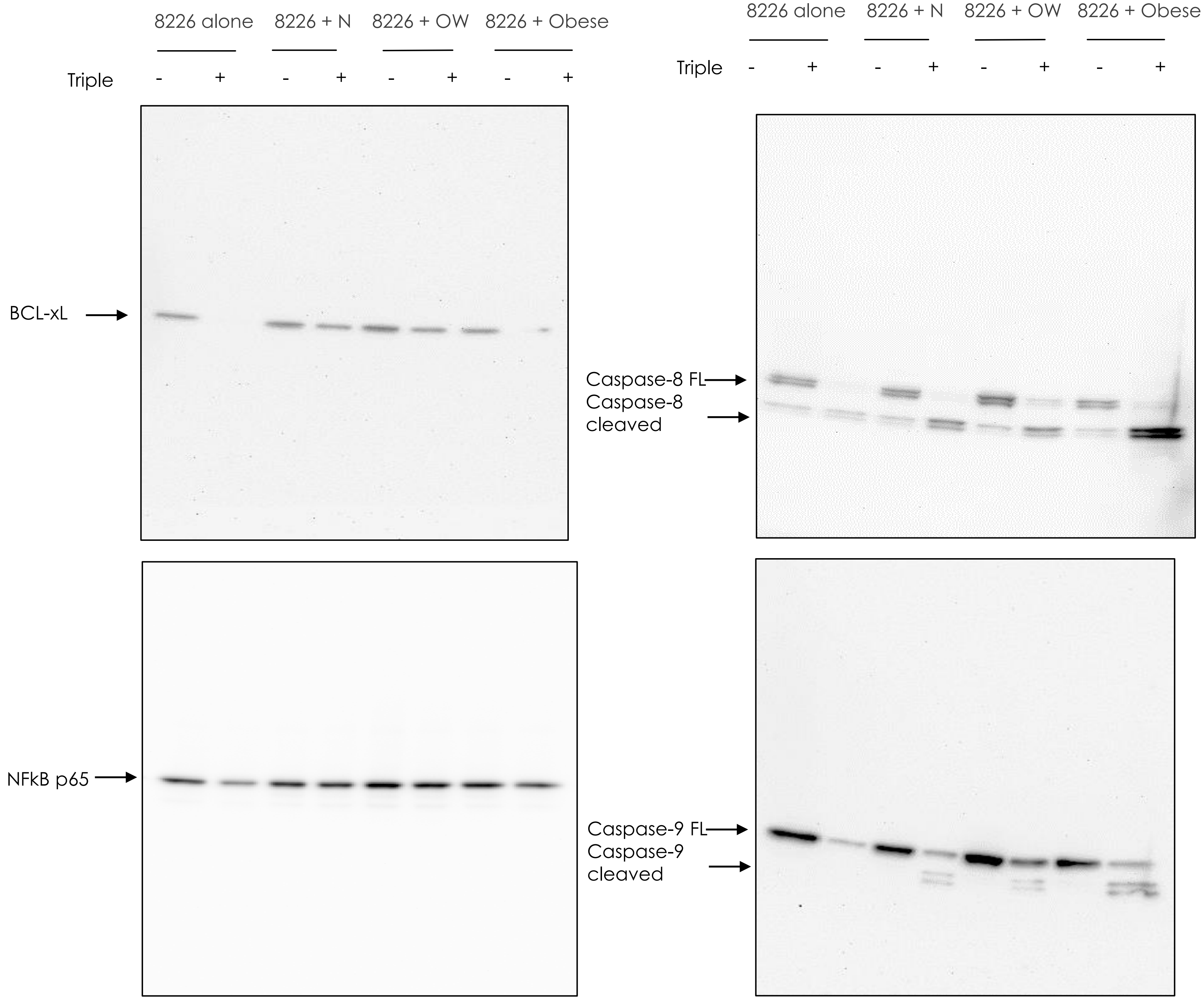

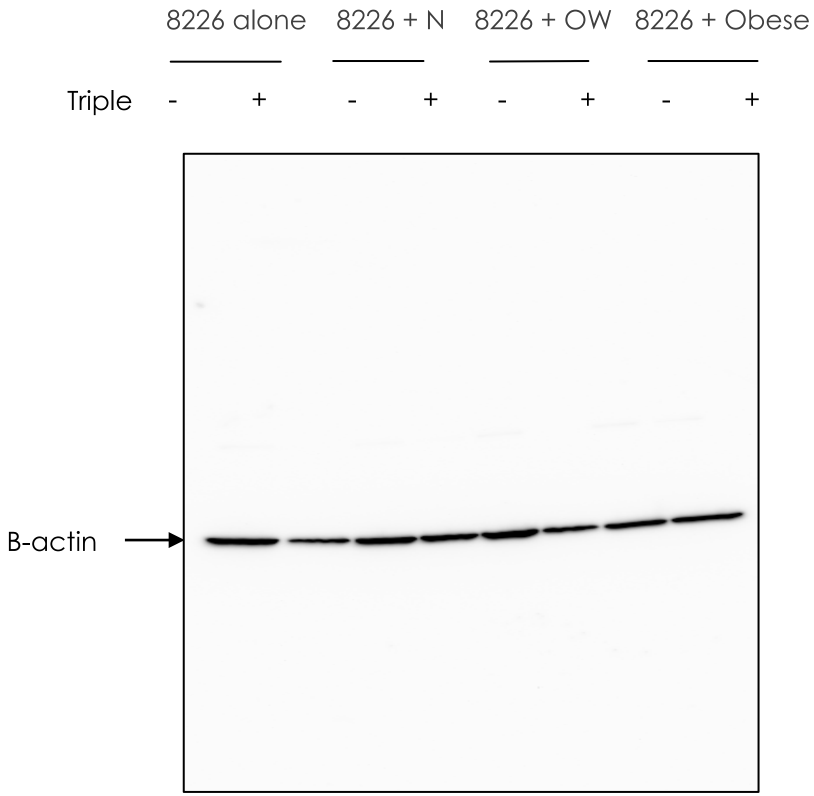

Figure S2. Uncropped WB images for Figure 2

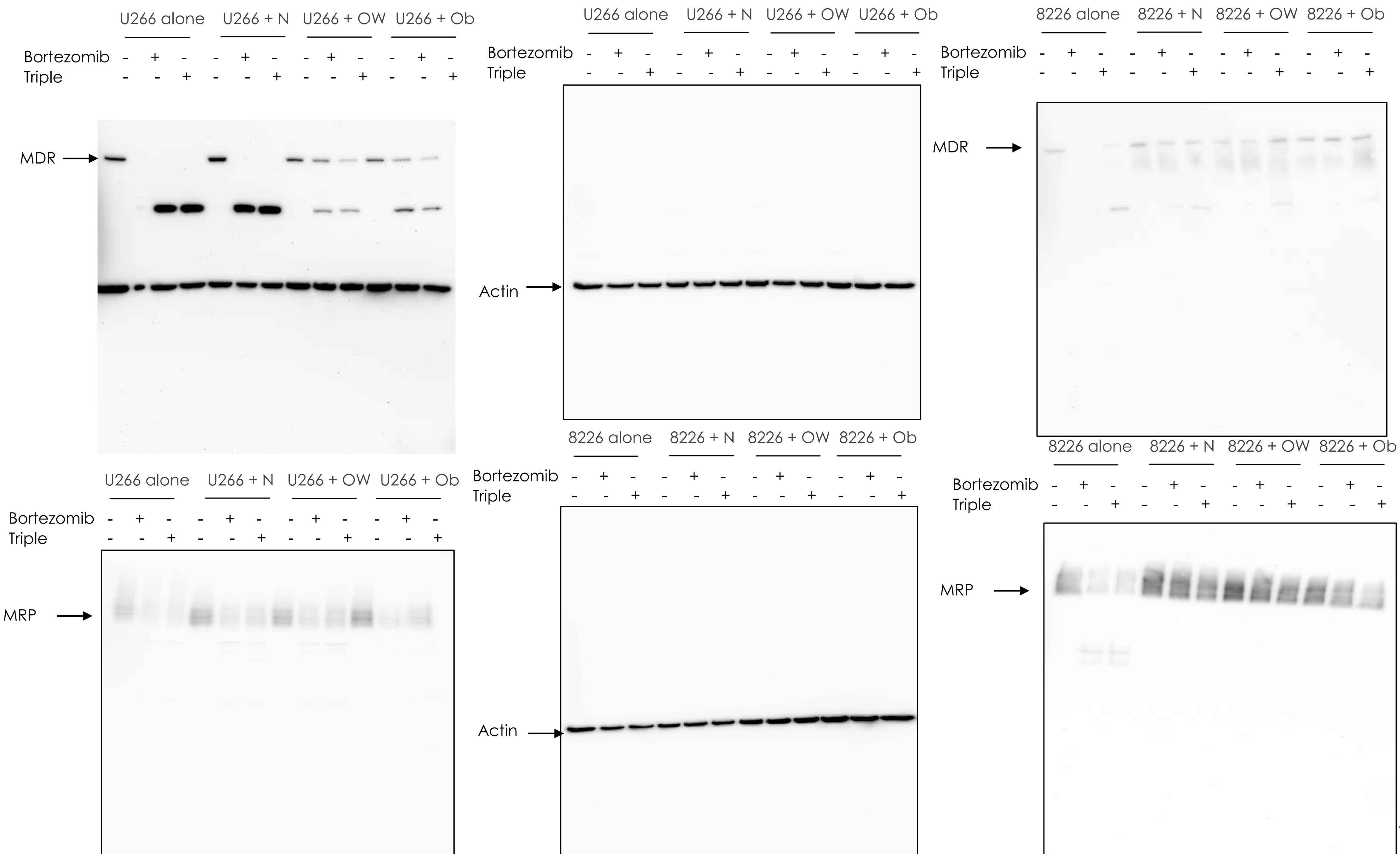

Figure S3. Uncropped WB images for Figure 3.

Normalized fold changes for All Western blots

Figure 1

Phospho-p65

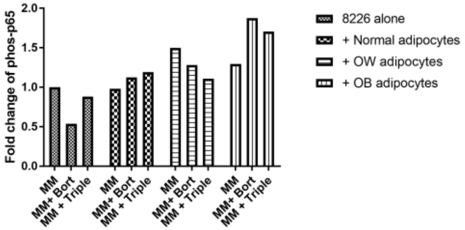

p65

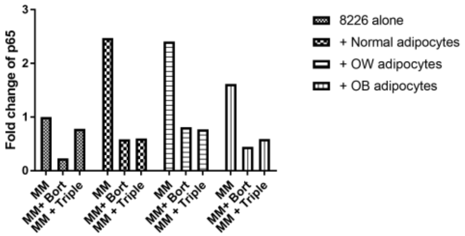

Cleaved Casp 3 (% Full length)

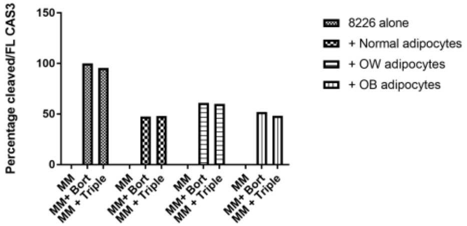

Figure 2A

BCL-xL

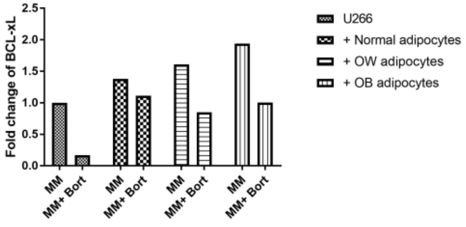

p65

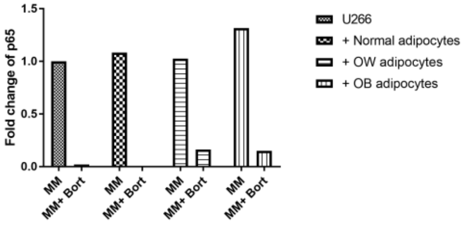

Cleaved Cas8

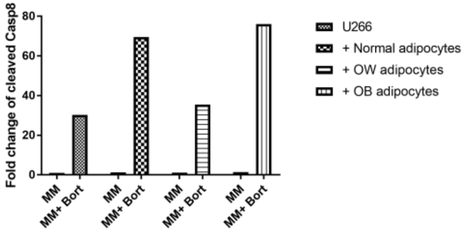

Cleaved Cas9

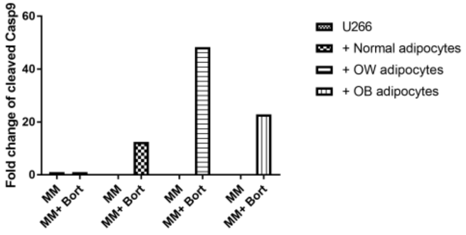

Figure 2B

BCL-xL

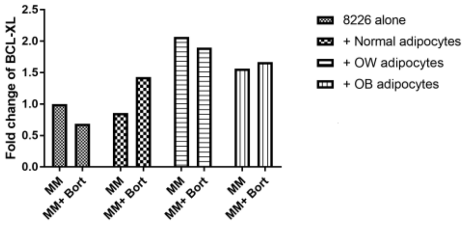

p65

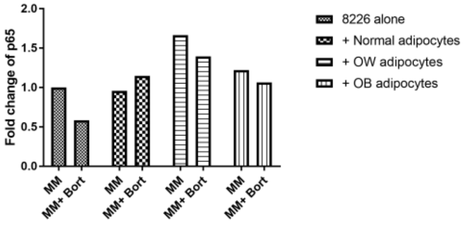

Cleaved Cas8

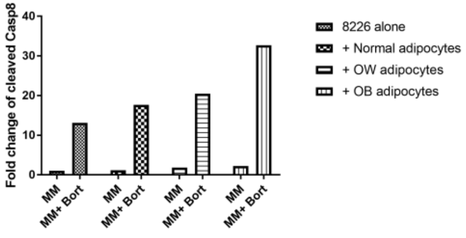

Cleaved Cas9

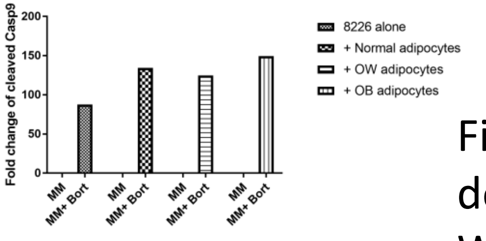

Figure S4. Normalized densitometry for all Western Blots

Normalized fold changes for All Western blots

Figure 2C

BCL-xL

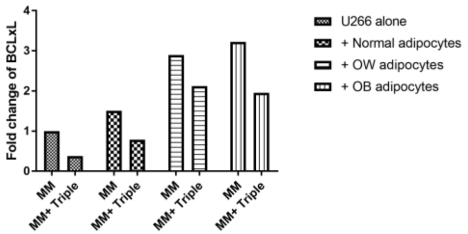

p65

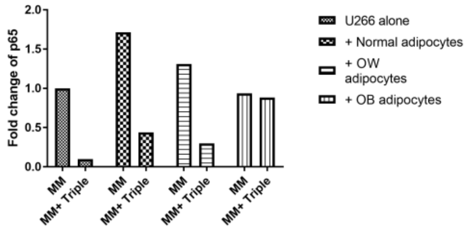

Cleaved Cas8

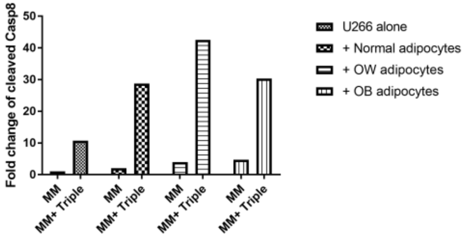

Cleaved Cas9

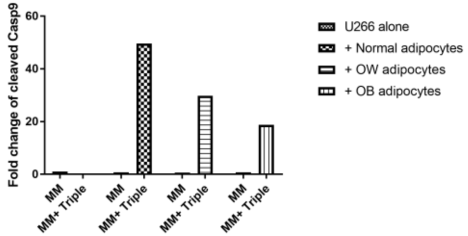

Figure 2D

BCL-xL

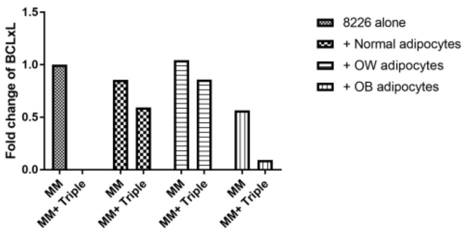

p65

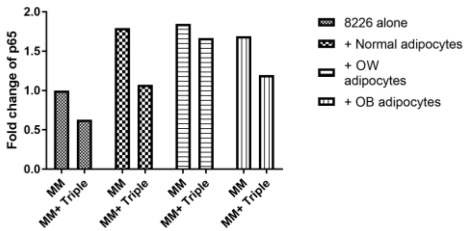

Cleaved Cas8

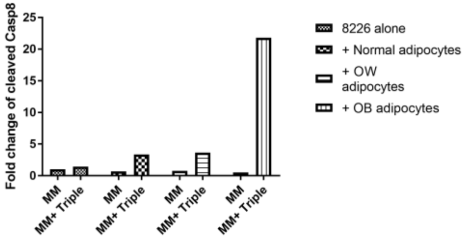

Cleaved Cas9

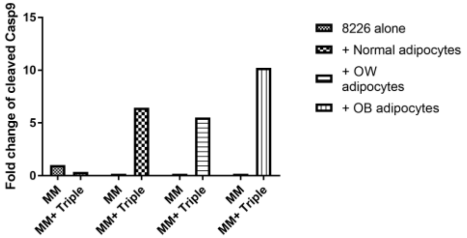

Figure 3

P-gp (U266)

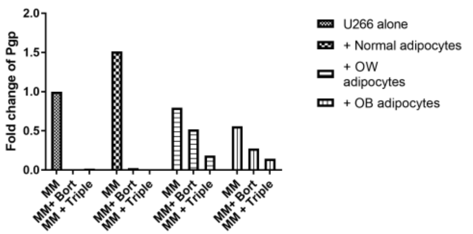

MRP (U266)

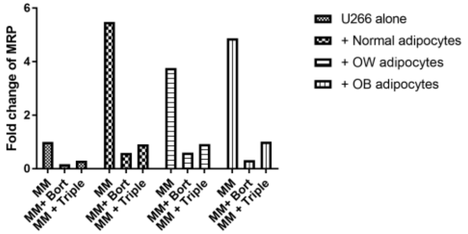

P-gp (8226)

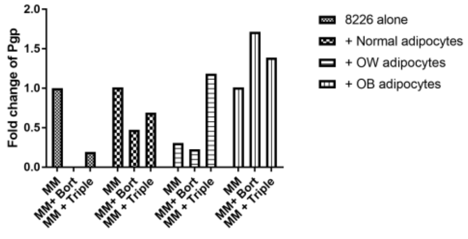

MRP (8226)

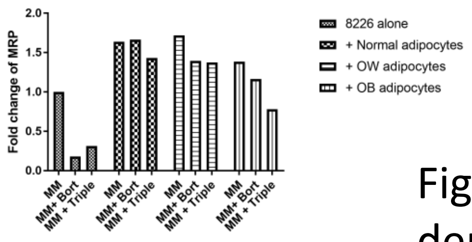

Figure S4. Normalized densitometry for all Western Blots

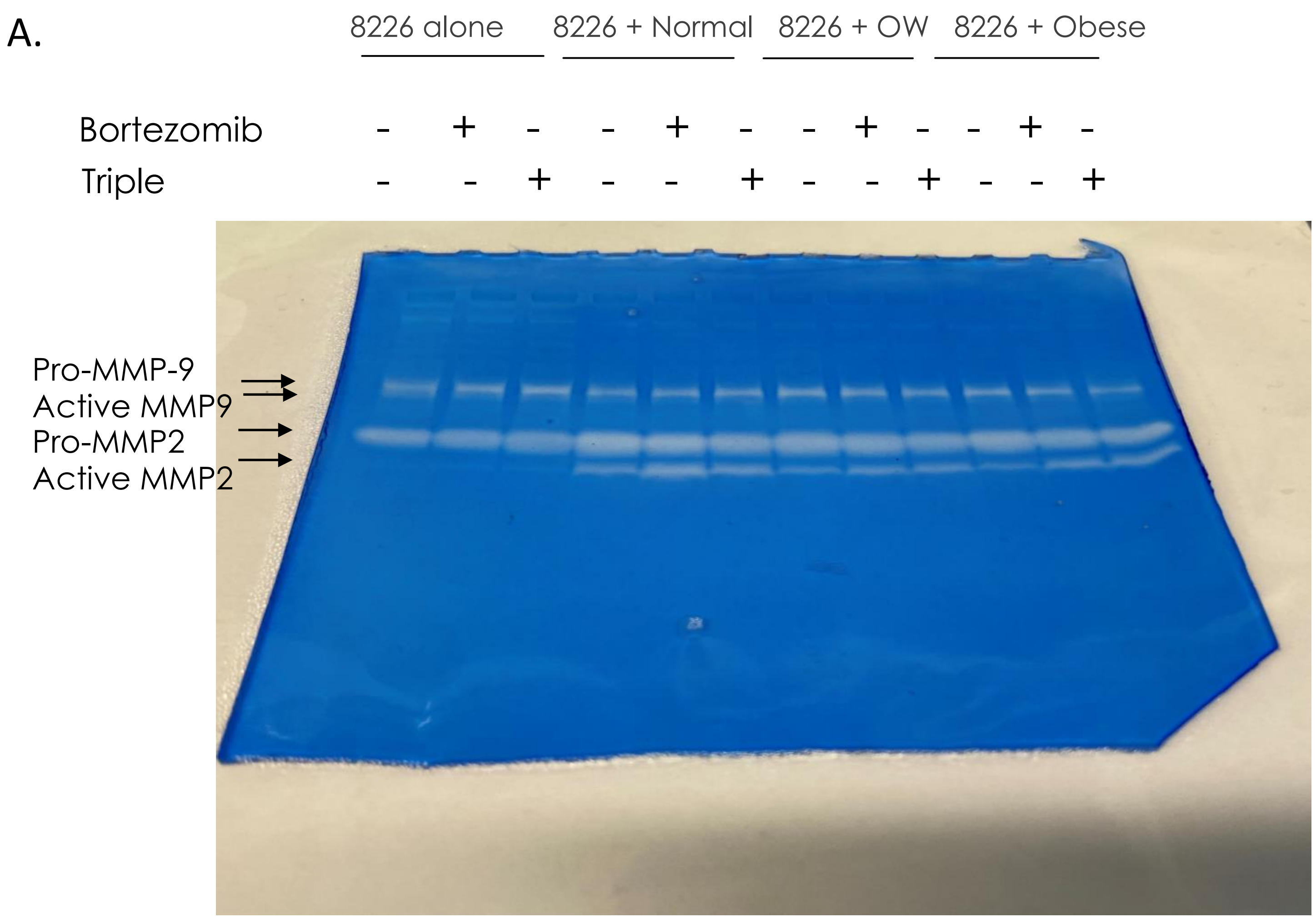

Figure S5: Uncropped zymogram

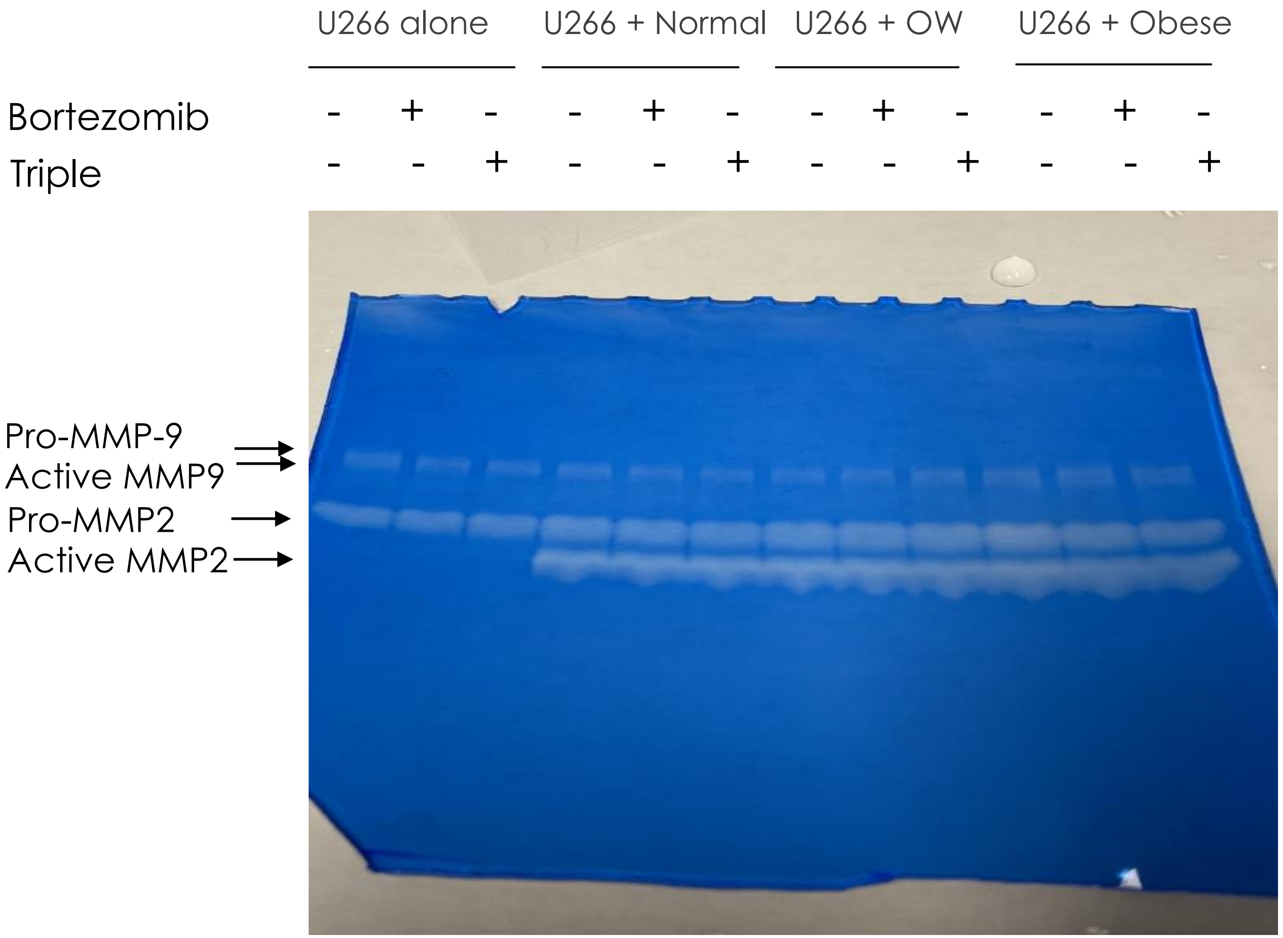

Figure S5. Uncropped zymogram
